# Advancing insect monitoring: analysis of mel-frequency cepstral coefficients from optical signals for body orientation estimation

**DOI:** 10.1007/s00340-026-08683-4

**Published:** 2026-05-26

**Authors:** Topu Saha, Benjamin P. Thomas

**Affiliations:** Department of Physics, New Jersey Institute of Technology, University Heights, Newark, NJ 07102 USA

## Abstract

Entomological photonic sensors enable continuous, automated and non-invasive monitoring of flying insects by recording optical signals of insects transiting in their field-of-view. These instruments can observe extremely large numbers of insects and provide ecological measurements such as aerial density [insect/m^3^] and biomass density [mg/m^3^] with temporal resolution down to a minute and minimal downtime. Nevertheless, their taxonomic resolution is limited; since deriving reliable taxonomic signatures from optical signals is difficult, knowing insects represent the most species-rich group of organisms. In this study, we focus on the influence of body orientation during transit, a factor that directly impacts how the body and wings contribute to the recorded signal. Using numerical simulations based on a 3D model of an *Apis mellifera* (honeybee), and laboratory experiments with *Musca domestica* (housefly), we analyze extinction signals using Mel-Frequency Cepstral Coefficients. They characterize the time-frequency structure of the waveform, caused by changes in orientation of an insect as it crosses the beam. A Gaussian Process regression model trained on these coefficients predicts symmetry-reduced orientation angles with high accuracy. By correcting for orientation, we can retrieve a more accurate estimate of the insect’s body optical cross-section. This correction may lead to improved identification and mass estimation. Our simulation results show that orientation effects can alter apparent cross-sections by up to 83% in this particular case, however this may be even more pronounced for insects with elongated bodies, underscoring the importance of accounting for them in biomass calculations obtained from photonic sensors. These findings demonstrate that orientation-sensitive signal analysis can refine predictor variables and improve the reliability of photonic sensors in providing ecological metrics beyond abundance, paving the way toward higher taxonomic resolution.

## Introduction

 Insects are the most diverse animal group on earth, and perform essential ecological functions, including pollination for plants, nutrient cycling through decomposition, regulation of food webs, serving as prey for numerous vertebrates and invertebrates [1–3]. Alongside these benefits, some insect species have negative impacts: agricultural pests can cause substantial crop losses, wood-boring species damage forestry resources, and many vectors transmit pathogens responsible for diseases like malaria, dengue, chikungunya, and Zika virus, causing thousands of deaths every year worldwide [4–7]. These complex dual roles underline the importance of understanding insect population dynamics for conserving beneficial species and managing those with negative effects. In addition, global reports of declining insect biomass, abundance, and diversity have intensified the need for accurate, high-resolution monitoring methods capable of capturing both yearly trends and daily/hourly behavioral responses [8–11]. Conventional insect monitoring methods like trap-based surveys offer high taxonomic resolution [12–16] but are labor-intensive, destructive, and often limited to low temporal resolution. In recent years, many new technologies, notably smart traps [17–21], computer vision and cameras [22–25], lidars [26–34], radars [35–38], and acoustic sensing technologies [39–42] have been put forward and have emerged as powerful complements to traditional approaches, enabling continuous, non-invasive detection of flying insects in their habitats. In this effort, our team at NJIT has developed a laser-based sensor called entomological bistatic optical sensor system (eBoss) which has demonstrated its ability for observing hundreds of thousands of insect transits over seasonal field deployment, revealing long-term aerial and biomass densities, daily activity peaks. These measurements have shown strong correlations with trap-based methods [43–49].

Entomological photonic sensors capture optical signals when insects fly in their field-of-view. These transit signals provide multiple optical signatures that may lead to various levels of taxonomic identification. Wingbeat frequency is by far the most common signature used for identification [50–52], as well as polarimetric and spectral signatures found in the body and wing cross-Sects [47, 53, 54]. In addition, the morphology and movement of wings, independently of their wingbeat frequency, may also be used for identification. Recent advances in insect flight research have been driven by the development and use of quantitative flow diagnostics, including digital particle image velocimetry and smoke flow visualization, leading to a better understanding of flight dynamics and wing movements [55–57]. However, when using entomological photonic sensors, the influence of the insect’s body orientation on the measured optical signals during its transit is often ignored entirely. Insect orientation can be expressed as pitch angle $\:\varphi\:$ (orientation of the body around its transversal lateral axis); yaw angle $\:\theta\:$ (orientation of the body about its vertical axis); and roll $\:\alpha\:$ (orientation of the body around its longitudinal axis), which characterize how the body is positioned with respect to the optical axis of the instrument. Variations in these angles alter the optical cross-section observed by the collecting optics of the instrument. While it does not impact the wingbeat frequency, it does change the amplitude and harmonic structure of the transit signal. Such effects can bias body and wing cross-section measurements, biomass estimates, polarization sensitivity, and spectral features. If ignored, these variations can reduce the accuracy of taxonomic classification and ecological inference from optical sensor data. Despite its importance, orientation is rarely measured or corrected in insect monitoring, largely because it is not directly observable in single-beam photonic systems.

This study intends to investigate the effect of the insect’s orientation on transit signals and presents a signal-processing based approach to estimate symmetry-reduced insect body orientation from single beam optical measurements. This work includes both a numerical simulation as well as a laboratory experiment using a transmission/extinction-based configuration, however we believe the outcomes of this study can be transposed to backscattered configurations (lidars). Our simulation used a 3D model of a flying bee to evaluate the influence of the yaw $\:\theta\:$ angle from 0 to 360° on the optical extinction signal. In addition, the experimental setup allows for the measurement of transit signals from flies (*Musca domestica*) while using a camera to monitor the insect’s orientation during its transit. Introducing Mel-Frequency Cepstral Coefficient (MFCC) analysis into the recorded transit signals offers a signal-processing pathway to detect orientation-related changes to improve optical signature measurements, biomass estimation, and the robustness of classification in single-beam photonic sensing.

## Mel-frequency cepstral coefficients (MFCCs)

The recorded signals are studied by measuring their Mel-Frequency Cepstral Coefficients (MFCCs). These coefficients were originally developed in the context of speech processing [58–62] as spectral shape descriptors. However, MFCCs are effective for detecting shape-related changes independent of fundamental frequency content, and their use for non-speech biological signal classification is well established. They have been successfully applied in bioacoustics and insect signal analysis [63–66], wingbeat classification [25, 67, 68], mosquito species identification from optical sensors [21, 69], acoustic recognition of singing insects across hundreds of species [64, 70], and animal vocalization analysis [71, 72]. MFCCs are also useful in medical image analysis, e.g. ECG diagnosis and chest X-ray classification [73, 74], palmprint recognition [75], and satellite image identification [76]. In this study, they capture variations in the extinction signal caused by a change in body orientation. The amplitude and harmonic structure in a wingbeat signal are influenced by wingbeat kinematics, the relative contributions of body and wing scattering, harmonic attenuation, and amplitude modulation that can change systematically with orientation. As MFCCs summarize this spectral shape, they are well suited for identifying orientation-dependent patterns within the extinction signal. To characterize the spectral content of each wingbeat transit signal beyond the fundamental frequency alone, we employ MFCCs as spectral envelope descriptors.

The computation proceeds through a sequence of well-defined operations on the measured optical cross-section signal σ(t), expressed in mm^2^. The power spectrum |σ(f)|^2^ is obtained via a Fast Fourier Transform, yielding spectral power density in mm^4^/Hz. This power spectrum is integrated across a set of approximately logarithmically spaced triangular frequency bands (the filterbank), producing band-integrated powers in mm^4^, each representing the total spectral energy within a defined frequency range. The power spectrum of each filterbank, S(k) can be computed as,

$$ {\mathrm{S}}\left( {\mathrm{f}} \right) = \left| {\sigma \left( {\mathrm{f}} \right)} \right|^{2} = \left| {\sum\nolimits_{{{\mathrm{t}}\_{\mathrm{min}}}}^{{{\mathrm{t}}\_{\mathrm{max}}}} {\sigma \left( {\mathrm{t}} \right).{\mathrm{e}}^{{ - 2\pi {\mathrm{if}}\;{\mathrm{t}}}} } } \right|^{2} $$

$$ {\mathrm{Considering}}\:{\mathrm{f}} = \frac{{\mathrm{k}}}{{{\mathrm{N}}.\Delta {\mathrm{t}}}}{\mathrm{and}},\:{\mathrm{t}} = {\mathrm{n}}.\Delta {\mathrm{t}}, $$

$$ {\mathrm{S}}\left( {\mathrm{k}} \right) = \left| {\sigma \left( {\mathrm{k}} \right)} \right|^{2} = \left| {\sum\nolimits_{{{\mathrm{n}}\_{\mathrm{min}}}}^{{{\mathrm{n}}\_{\mathrm{max}}}} {\sigma \left( {\mathrm{n}} \right).{\mathrm{e}}^{{\frac{{ - 2\pi {\mathrm{ink}}}}{{\mathrm{N}}}}} } } \right|^{2} $$

Where N is the total number of bins, k represents frequency bin index (k = 0,1,2,3…,N-1) of the n-th sample with time step $\:\varDelta\:\mathrm{t}$. The frequency components (f) in the power spectrum are converted into mel-scale (m) using the formula:

$$ {\mathrm{m}} = 2595\:{\mathrm{log}}_{{10}} \left( {1 + \frac{{\mathrm{f}}}{{700}}} \right) $$

A transfer function, $\:{\mathrm{H}}_{\mathrm{m}}\left(\mathrm{k}\right)$ is computed for each of the m-th filter following the conditions below [73]:

$$ {\mathrm{H}}_{{\mathrm{m}}} \left( {\mathrm{k}} \right) = \left\{ {\begin{array}{*{20}c} 0 & {{\mathrm{k}} < {\mathrm{f}}({\mathrm{m}} - 1)} \\ {\frac{{{\mathrm{k}} - {\mathrm{f}}({\mathrm{m}} - 1)}}{{{\mathrm{f}}\left( {\mathrm{m}} \right) - {\mathrm{f}}({\mathrm{m}} - 1)}}} & {{\mathrm{f}}\left( {{\mathrm{m}} - 1} \right) \le {\text{k < f}}\left( {\mathrm{m}} \right)} \\ 1 & {{\mathrm{k}} = {\mathrm{f}}\left( {\mathrm{m}} \right)} \\ {\frac{{{\mathrm{f}}\left( {{\mathrm{m}} + 1} \right) - {\mathrm{k}}}}{{{\mathrm{f}}\left( {{\mathrm{m}} + 1} \right) - {\mathrm{f}}\left( {\mathrm{m}} \right)}}} & {{\mathrm{f}}\left( {\mathrm{m}} \right) < {\mathrm{k}} \le {\mathrm{f}}\left( {{\mathrm{m}} + 1} \right)} \\ 0 & {{\mathrm{k}} > {\mathrm{f}}\left( {{\mathrm{m}} + 1} \right)} \\ \end{array} } \right. $$

where $\:\mathrm{f}\left(\mathrm{m}\right)$ is the center frequency bin index of the triangular filter. The approximately logarithmic spacing of the filterbank is particularly well suited to harmonic signals: since the harmonics of the wingbeat fundamental (f_0_, 2f_0_, 3f_0_, …) are linearly spaced in frequency, a logarithmic filterbank allocates comparable resolution to each harmonic, providing balanced coverage of the physically meaningful spectral content.

**Fig. 1:**
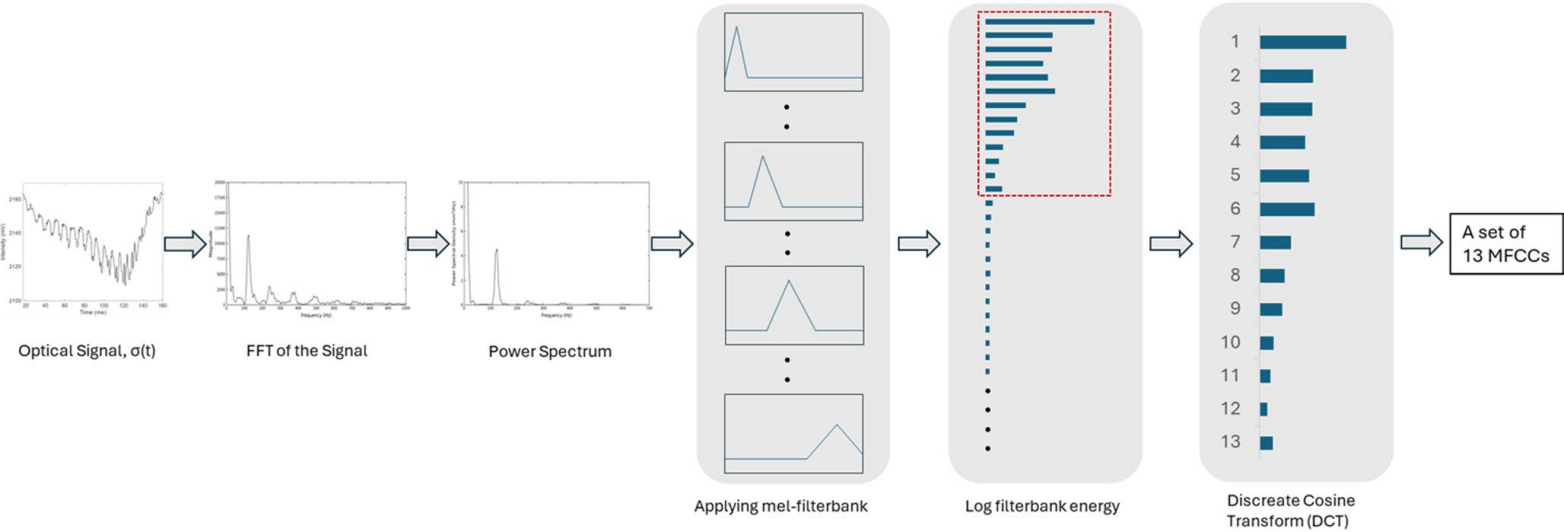
Flowchart of the MFCC features extraction process from optical signal

A logarithmic compression is then applied to the band energies, ${\mathrm{E}}_{{\mathrm{m}}} \left( {\mathrm{k}} \right) = {\mathrm{log}}\left[ {\sum {{\mathrm{S}}\left( {\mathrm{k}} \right).{\mathrm{H}}_{{\mathrm{m}}} \left( {\mathrm{k}} \right)} } \right]$. Taking logarithm of a dimensional quantity is understood as an implicit normalization: the physically meaningful content lies in the relative distribution of energy across bands rather than in absolute values. The resulting log-filterbank energies are therefore dimensionless. Finally, a Discrete Cosine Transform (DCT) is computed across the ordered set of log-filterbank energies, decomposing the shape of the log-spectral envelope into a series of cosine basis functions, producing a set of 13 coefficients. A flowchart of the MFCC feature extraction process is shown in Fig. 1.

The zeroth coefficient represents the overall signal level, the first coefficient captures the broadest spectral tilt, and successive coefficients describe increasingly fine features of the spectral shape. Since DCT is a linear operation on dimensionless inputs, the resulting cepstral coefficients are themselves dimensionless, hence a physical unit is absent on the MFCC axes in Figs. 4 and 9. Physically, these coefficients encode how energy is distributed across the harmonics of the wingbeat, which corresponds to the timbre of the wingbeat, the quality that distinguishes two insects with the same fundamental frequency but different wing morphology or kinematics.

The first 13 coefficients are extracted from each optical signal using a built-in library in python. The lower-order coefficients capture the dominant spectral envelope shape while higher-order coefficients primarily encode fine spectral detail susceptible to noise, coefficients past the first 13 do not improve classification accuracy and are therefore ignored. Previous studies in insect classification, bioacoustics, and medical signal analysis also indicate that the first 12–13 MFCCs are sufficient to preserve relevant information [21, 77–79].

## Numerical simulation

To examine how an insect’s body orientation affects transit signals, a numerical simulation was implemented in Blender 4.3 environment. The aim of the simulation is to investigate how the intensity of transmitted light varies as a function of different yaw angles, and to confirm their potential correlation with MFCCs.

### Simulation model

**Fig. 2:**
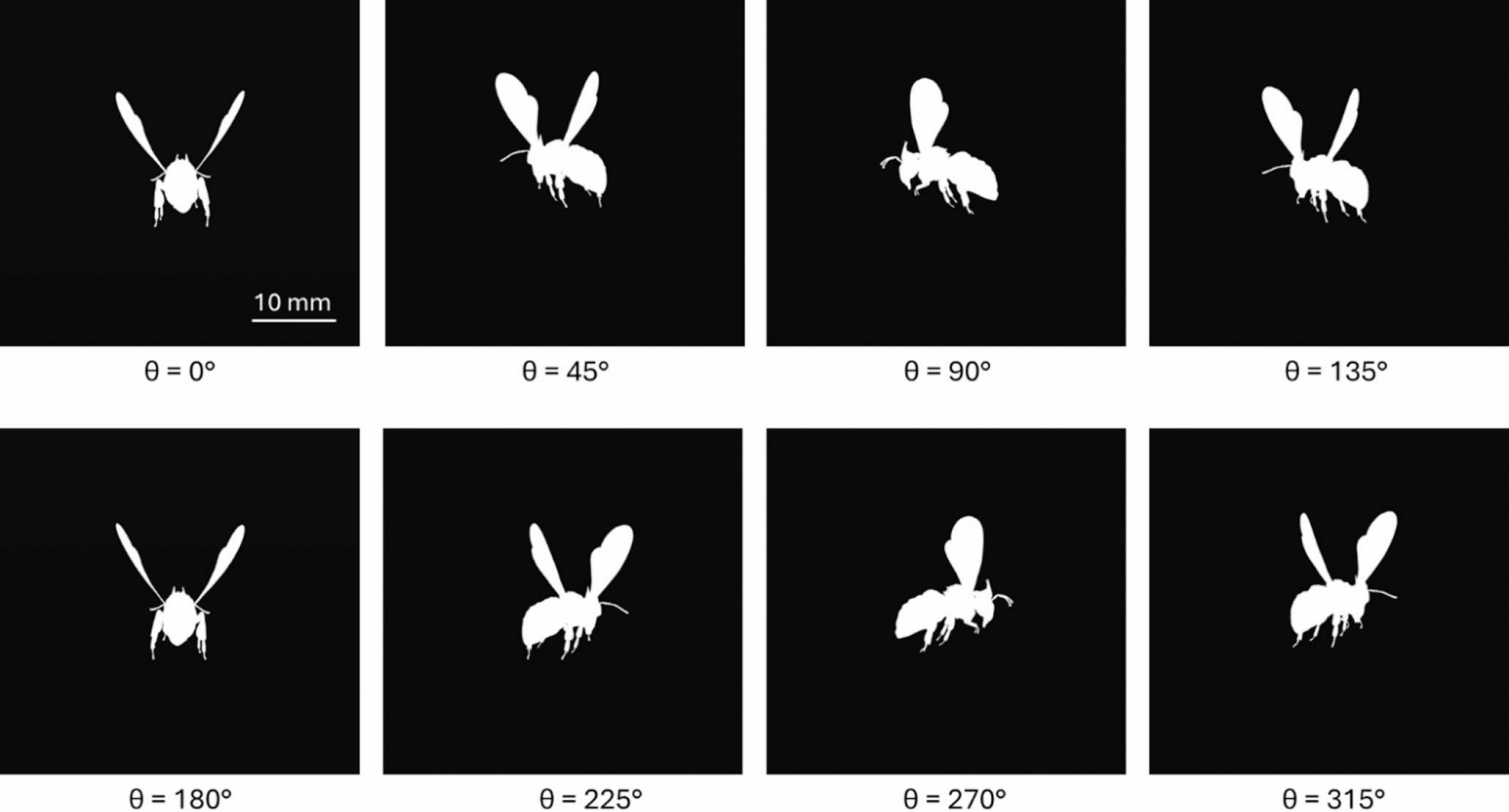
Rendered images of the flying insect at different yaw angles ($\:\theta\:$). Complementary angles (0 and 180°, 45 and 225°, 90 and 270°, 135 and 315°) show nearly identical projections

A detailed 3D insect model was imported into Blender software from an open-source repository and modified with animations to reproduce realistic flight behavior. Wingbeat motion is implemented using keyframe animation in Dope Sheet and Graph Editor, allowing the generation of 100 wing positions to describe a full wing cycle. The wing motion is designed to mimic natural flight dynamics, with adequate amplitude and timing to produce periodic optical fluctuations. A virtual camera is positioned at a fixed distance from the animated insect and set to ‘orthographic’ projection mode to simulate collimated, parallel rays, ensuring that object size and projected area remain constant regardless of its depth. Using a custom Python script written in Blender’s API, the camera is programmatically rotated in the horizontal plane in steps of 5°, completing a full 360° cycle to replicate the equivalent effect of changing yaw angles due to body rotation of a flying insect within a fixed laser beam. The rendering process is configured to output high-contrast black and white images for each frame. In these images, the insect body and wings appear in solid white, representing the regions that would absorb or scatter incident light. The background is rendered in black, corresponding to areas where light is fully transmitted. This allows a straightforward approach for the estimation of insect’s optical cross-section by simply counting the number of white pixels per frame. Figure 2 shows example renderings of the flying insect at various yaw $\:\theta\:$ angles, where orientation-dependent variation in projected area is clearly visible.

**Fig. 3:**
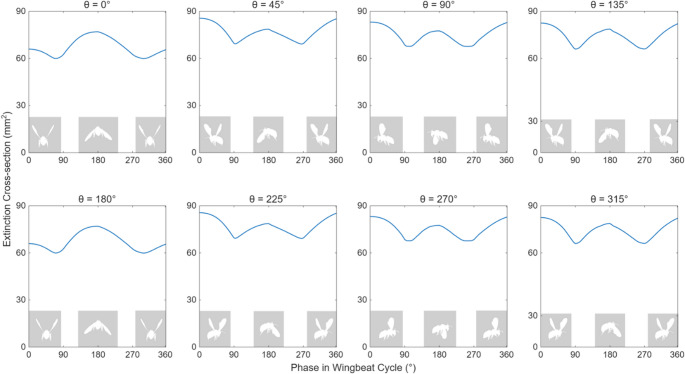
Simulated transit signals for a single wingbeat cycle, computed from the rendered images at different yaw angles ($\:\theta\:$). The x-axis of the plots indicates different phases in a wingbeat cycle, and the y-axis indicates the extinction cross-section. The shaded images on the plots show variations in wing positions at 0,180, and 360° phase angles

The images rendered at different yaw angles are analyzed to simulate transit signals that would be recorded by a detector; assuming a flying insect remains entirely on the spatially homogeneous laser beam and the cross-section of the laser beam ($\:{\sigma\:}_{L}$) is larger than the cross-section of the insect ($\:{\sigma\:}_{I}$). For simplicity, the insect is considered to be perfectly opaque (transmission = 0), meaning that $\:{\sigma\:}_{I}$ is equal to the projected area of the insect. The extinction cross-section varies over time due to the flapping of the insect’s wings and different body orientations. Figure 3 presents extinction signals generated at different yaw angles, illustrating how the amplitude and shape of the waveform changes at different phases in a wingbeat cycle.

### Simulation results

To evaluate the relationship between insect’s body orientation (yaw angle) and optical signal characteristics, MFCCs and optical body cross-sections ($\:{\sigma\:}_{B}$) are extracted from the simulated transit signals and image analysis. Later, a Gaussian Process regression model is trained using all the MFCC features to predict yaw angles.

**Fig. 4:**
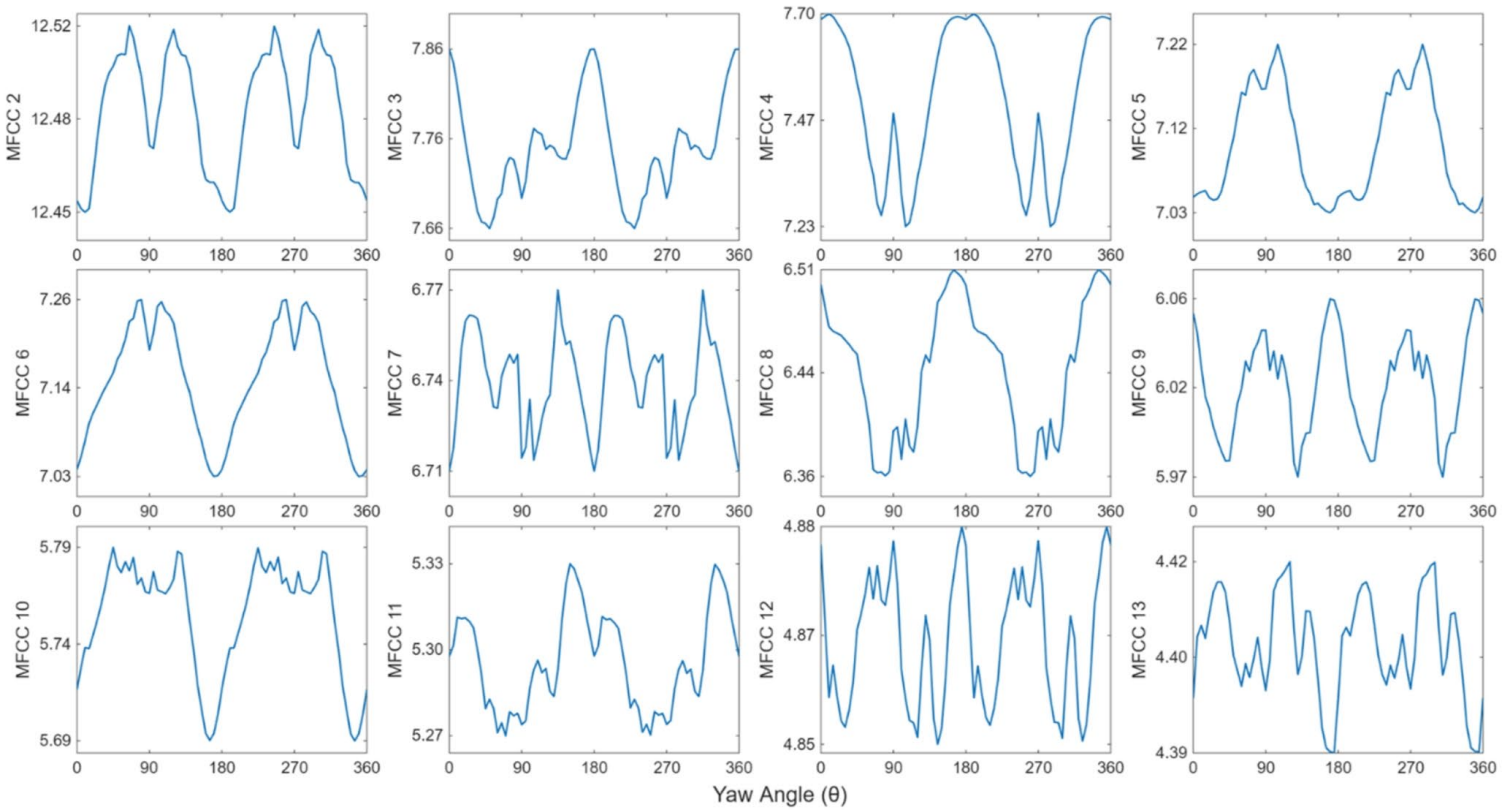
Variation of MFCC coefficients (2nd − 13th ) as a function of yaw angles, symmetry is observed beyond $\:\theta\:=180^\circ\:.$

Using ‘librosa’ library in Python, the first and fundamental 13 MFCCs are computed for all the signals, the first coefficient returns the log energy of the spectral envelope while the rest (MFCC 2 – MFCC 13) describe signal fluctuations. Figure 4 shows the change in each of the coefficients separately as a function of yaw angle over a full cycle in the 0 to 360° range. Among them, several coefficients show consistent patterns as the yaw angle changes. Results in the figure show several MFCC coefficients (3,4,8 and 9) exhibit maximal values near 0, 180, and 360° (when the flying insect’s head or tail faces along the direction of optical axis), while other coefficients (2,5,6) generate peaks near 90 and 270° (insect’s body alignment is sideways to the optical axis). The results clearly demonstrate that MFCCs are changing with orientation, whereas yaw angles separated by 180° produce symmetrical optical responses.

**Fig. 5:**
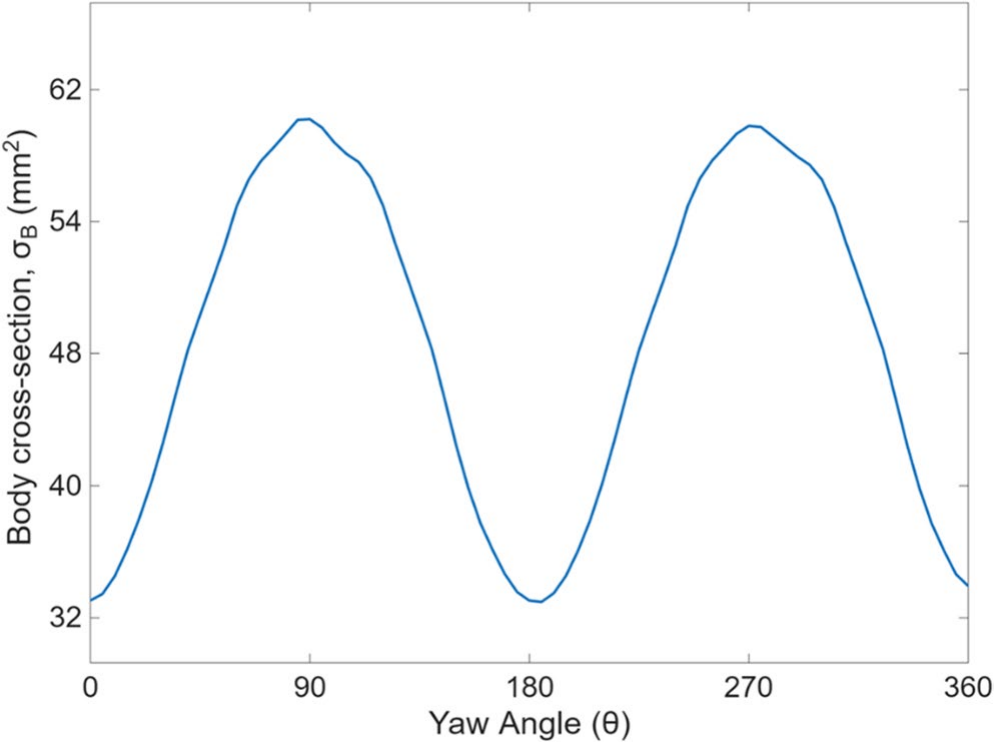
Variation in the insect’s body cross-section as a function of yaw angle

The extinction cross-section of the insect’s body is also measured from the simulated images for all the available yaw angles. Figure 5 shows a clear position-dependent pattern of the body cross-section. As the yaw angle increased from 0 to 90°, the projected cross-section gradually increased. Beyond this point, the cross-section decreases until about 180° and repeated afterwards. The apparent size of the insect body, as seen along the optical axis, increased by 83% relative to its minimum value, which signifies the effect of body orientation in shaping the optical signal. Additionally, the clear symmetry observed in the measurements reinforces the assumption that yaw angles between 0 and 180° are equivalent to those between 180 and 360°, which enables angular space reduction in modeling or learning tasks.

The results demonstrate that MFCCs and body cross-section are changing with the orientations of the insect. The projected cross-section of an insect’s body is symmetric about multiple axes, causing the optical signature to be repeated at regular rotational intervals. As shown in the analysis, body orientations separated by 180° produce equivalent extinction patterns, making them impossible to differentiate with a single-beam photonic system. Also, the body orientation at an angle $\:\theta\:\ge\:90^\circ\:$ is indistinguishable from that at (180°– $\:\theta\:$), as it mirrors the optical responses. By mapping these complementary angles into a single 0 to 90° range, we remove redundancy and define a fundamental orientation domain that captures all unique optical configurations.

Based on the orientation dependence, and symmetry observed in the simulation results, we predict the insect yaw angle, $\:\theta\:$ using a Gaussian Process Regression model. The model is trained with all 13 MFCC features and evaluated with a 5-fold cross-validation method to ensure that all datapoints contribute to both training and testing rather than a single train–test split. Within the symmetry-reduced range of 0–90°, the model shows strong agreement between predicted and measured orientations, with $\:{R}^{2}=0.99$, an average relative error of 2.8%, and root-mean-square error (RMSE) of 0.83. The numerical simulation effectively captures the MFCC responses to body orientation, demonstrating its predictive capability.

## Laboratory experiment

### Experimental setup

**Fig. 6:**
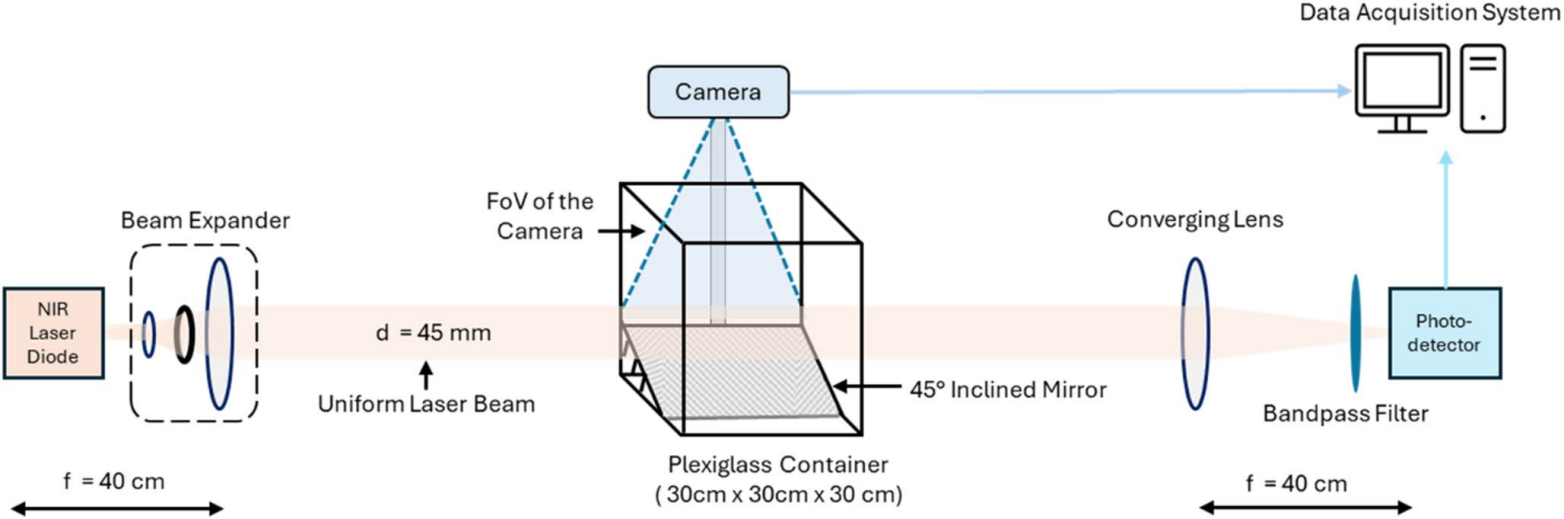
Schematic of the experimental setup showing the eBoss optical sensor, high-speed camera, and insect flight container

An experimental setup is employed to capture transit signals in a transmission configuration while simultaneously monitoring the orientation and trajectory of the insect during its transit using a video camera [80, 81]. This setup reproduces the principle of operation of our field deployable Entomological Bistatic Optical Sensor System (eBoss) [43, 44, 46], while also adding the possibility of recording videos of the insects during its flight. Figure 6 shows the optical layout of this experiment. The laser source is a 5 mW continuous-wave (CW), near infrared (NIR) laser diode (980 nm, CPS980, Thorlabs, USA). A beam expander system, containing two diverging lenses and a pinhole, is used to expand the original laser beam and allow the central portion of the beam to pass, maintaining uniform density (flat-top approximation). As a result, the diameter of the beam reaches 45 mm and propagates horizontally towards the receiver. At the end of the optical path, the light is collected by a converging lens of 400 mm focal length, goes through a spectral bandpass filter, and focuses onto the active area of a silicon amplified photodetector (PDA36A2, Thorlabs, USA). The bandpass filter transmits above 95% of light from 950 to 1000 nm wavelength to reduce the effect of unwanted light sources. The detector has an effective bandwidth of 90 kHz and has an active area of 3.6 × 3.6 mm. The optical signal is recorded at a sampling frequency of 30,517 Hz using a 16-bit digitizer (M4i4420- × 8, Spectrum, USA) with a 5 V range. The acquisition system is integrated into a regular desktop computer for continuous data collection. A transparent plexiglass container (30 × 30 × 30 cm) is placed in the middle of the optical path to enclose the flying insect. The insect is a *Musca domestica*, this species was chosen as they are easy to rear and remain very active even when trapped in a container, only one insect is introduced at a time to avoid multiple insects transiting at the same time. The flight speed of a housefly is approximately 2 m/s, yielding a minimum transit time of 25 ms for a straight trajectory perpendicular to the beam. In practice, however, such perpendicular crossings are rare; most transits involve curved or oblique flight paths with a significant component along the beam axis, resulting in longer transit times and an average transit time of approximately 75 ms. A planar mirror is positioned at 45° inclination relative to the bottom surface, enabling the camera to capture the direct view and reflected view of the flying insect in the same frame, allowing to retrieve the x, y and z coordinates of the insect. The high-speed digital camera (USBFHD08S-MFV, ELP, China) is mounted at the top of the container, looking downward, to capture videos up to 120 frames per second (fps) at 480p, allowing detailed information of flight trajectories of the insect. The focal length of the camera lens is adjustable from 5 to 50 mm, enabling a wide field of view within a short distance. A continuous-wave (CW) studio light (Raleno, China) is used for consistent and uniform illumination to enhance the video quality and to minimize motion blur during recording. The camera system is connected via a USB cable to the computer to save recorded videos.

### Data analysis

**Fig. 7:**
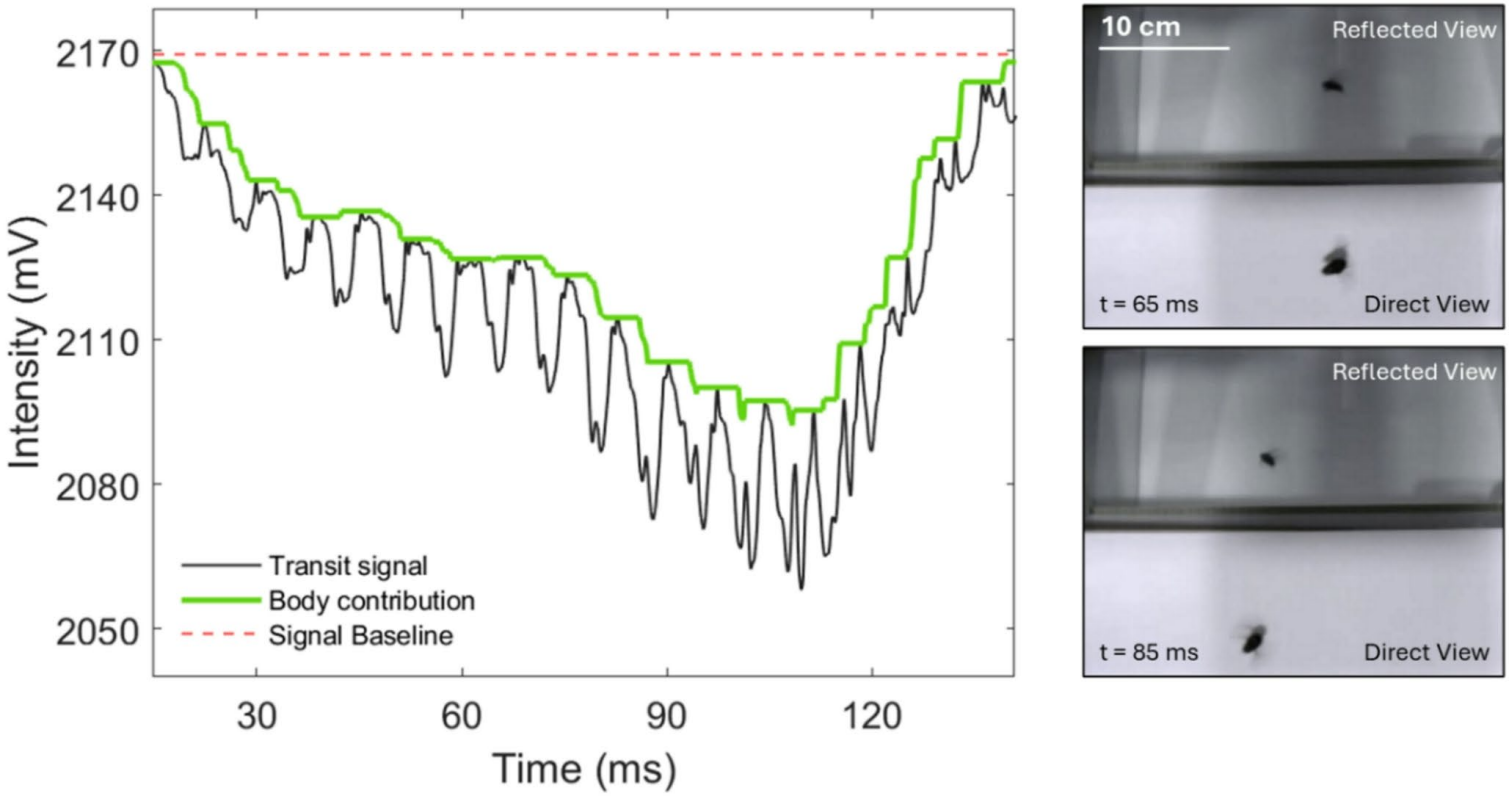
Left: example of an insect transit signal when it crosses the probe volume of the laser beam; Right: two video frames, each frame shows the direct view (bottom part) and reflected view (top part) allowing to track the insect position in 3 dimensions

The data acquisition system simultaneously records optical transit signals and videos from the detector and camera respectively. A Python script is continuously running in the ‘VS Code’ environment that detects and saves an insect flight when it passes through the field of view of both systems (detector and camera). A time-synchronized command in the coding script ensures proper alignment between video frames and transit signals. Figure 7 shows an example transit signal and two of the corresponding video frames.

As shown in the figure, the sharp and periodic drops in the recorded signal are caused by the rapid movement of insect wings, and the gradual, slow-changing envelope (green-colored) is due to the body contribution. Throughout the entire transit time, the insect may change its body orientation, potentially impacting the signal spectral features. Therefore, a transit signal is segmented into multiple portions corresponding to each video frame available. The body optical cross-section can be retrieved from the segmented transit signals, while the video frames provide orientation-related information.

#### Measurement of body cross-section

**Fig. 8:**
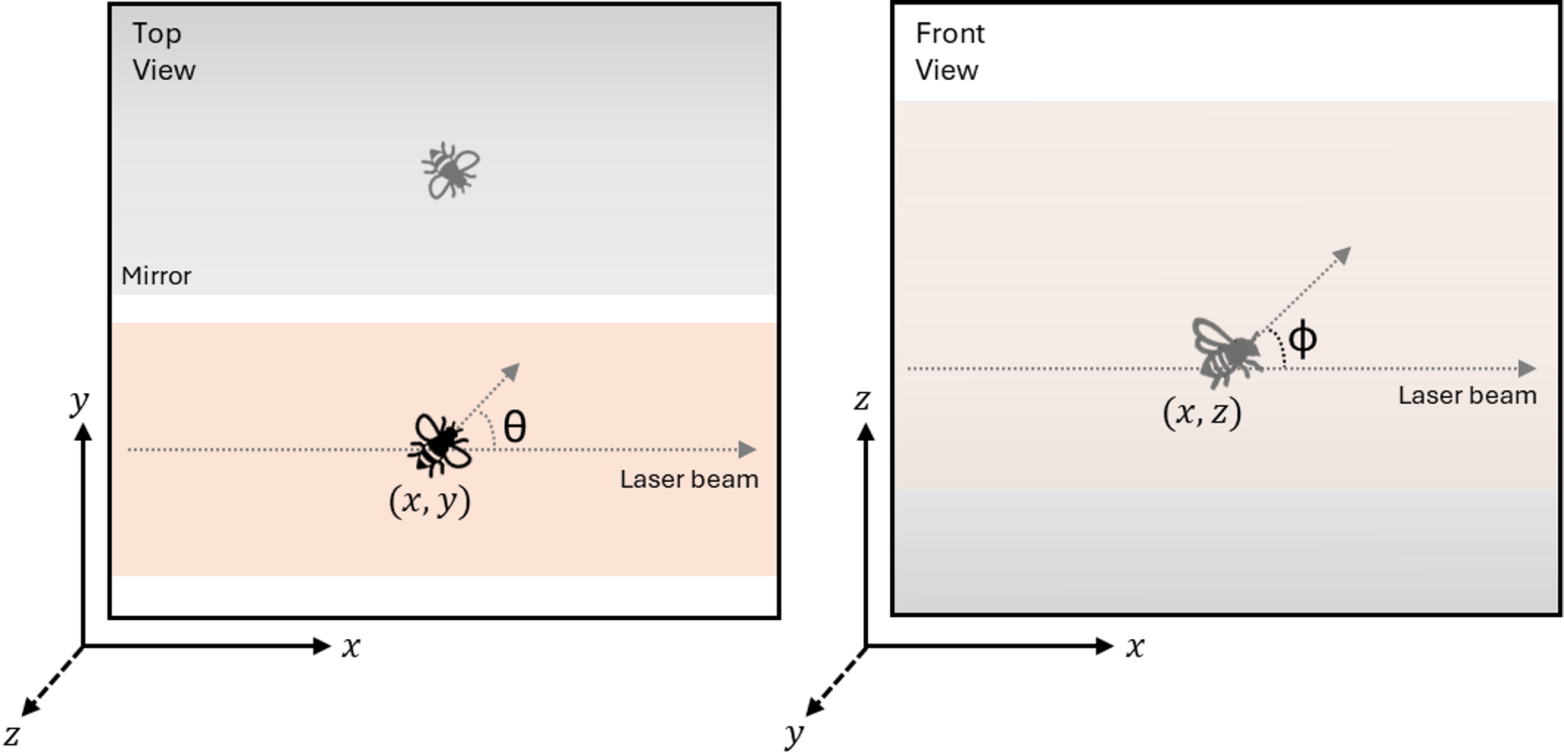
Top view (camera view) and front view of the system, showing how spatial coordinates ($\:x,y,z$) and body orientation angles ($\:\theta\:$, $\:\varphi\:$) are measured

Optical extinction cross-section is characterized by the signal drop compared to the baseline value defined in the absence of any target inside the laser beam. As the laser spatial profile is assumed to be uniform, and the cross-section of the laser is known, the baseline voltage and signal drops can be expressed in mm^2^ for measuring the body cross-section by using the following equation:

$$\:{\sigma\:}_{B}={\sigma\:}_{pv}\:.\frac{{I}_{0}-{I}_{B}}{{I}_{0}}$$

where $\:{\sigma\:}_{B}$ is the insect’s optical extinction cross-section (mm^2^) for a segmented signal at $\:\theta\:$ and $\:\varphi\:$ degrees body orientation, $\:{\sigma\:}_{pv}$ is the cross-section of the laser beam in mm^2^, $\:{I}_{0}$ is the value of the baseline voltage, and $\:{I}_{B}$ is the intensity (V) of the signal attenuation due to body orientation as shown in Fig. 8.

#### Measurement of position and angles

The position and orientation of flying insects can be extracted from high-speed video recordings captured at 120 frames per second. Each frame is analyzed using an image processing toolbox to obtain both spatial coordinates (x, y,z) and body orientations (yaw, θ and pitch, φ angles), based on the direct and reflected view of the insect. A schematic of the system is shown in Fig. 8 from top and front viewpoints, where the laser beam propagates along the horizontal x-axis while the y-axis is defined as perpendicular to the x-axis on the horizontal plane. A planar mirror is positioned behind (front-view) the laser beam, and provides a reflected view of the insect, essential for estimating its vertical position z-axis. In addition to position, the orientation of the insect in 3D space is partially reconstructed. The insect’s body axis is annotated for each frame within the field of view of the laser beam to derive pitch and yaw angles. The yaw angle, θ is defined as the angular deviation of the body axis (direct view) on the horizontal plane (xy plane) and is calculated from the angle between optical path (x-axis) and the body axis projection on the xy plane. The pitch angle, φ is defined as the angle between the insect’s body axis (from the reflected view) and the direction of the laser beam on the vertical plane (xz plane), representing the inclination of the body along the optical axis. The roll angle (α), does not have any significant impact in projected cross-section, and thus the effect of roll is ignored for further analysis.

### Experimental results

Using the laboratory setup, a total of 637 transit signals has been recorded and analyzed, providing a total of 1876 individual segments (frame and the corresponding part of the transit signal). Optical body extinction cross-sections ($\:{\sigma\:}_{B}$), 13 MFCCs, pitch ($\:\varphi\:$) and yaw ($\:\theta\:$) angles are retrieved for all the segmented signals. As the simulation results indicate, the extinction cross-sections exhibit position-dependent symmetry, and thus can be mapped into a reduced equivalent range of 0 to 90°. To achieve this, all experimentally measured angles (both $\:\theta\:$ and $\:\varphi\:$) are converted into 0–90° to investigate symmetry-reduced body orientation effects. The angles and measured parameters (MFCCs and body extinction cross-sections) are then averaged in 5° bins to reduce the impact of small fluctuations in the measured angles. This transformation improves statistical robustness by aggregating equivalent measurements as well as minimizing angular variations.

#### MFCC variation with body orientation

**Fig. 9:**
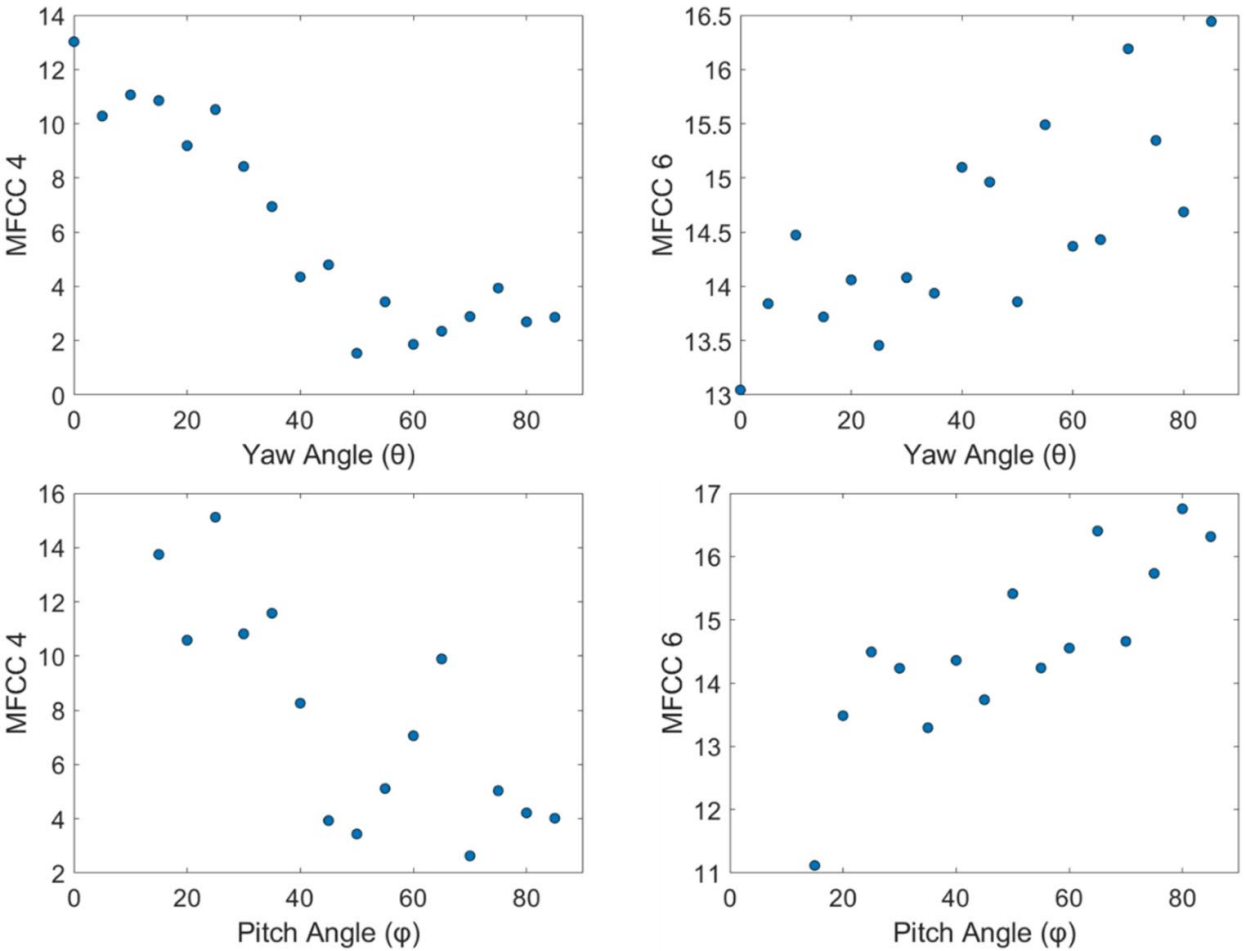
Selected MFCCs as a function of symmetry-reduced body orientation: yaw angle ($\:\theta\:$) in the top and pitch angle ($\:\varphi\:$) in the bottom

All 13 MFCCs are computed from the segmented transit signals, and each of them exhibit correlation with both pitch, $\:\varphi\:$ and yaw, $\:\theta\:$ angles. While some of the coefficients, e.g., MFCC 4 and MFCC 6 are better sensitive to orientation angles, a few of them are weakly correlated. But all the coefficients contribute effectively to training the model for body orientation estimation. As shown in Fig. 9, MFCC 4 has a decreasing trend as $\:\theta\:$ and $\:\varphi\:$ increase, and MFCC 6 gradually increases as both angles increase. These orientation dependent patterns confirm that MFCC features are sensitive to both angular components, providing valuable information for orientation estimation in free-flight measurements.

#### Optical cross-section variation with body orientation

**Fig. 10:**
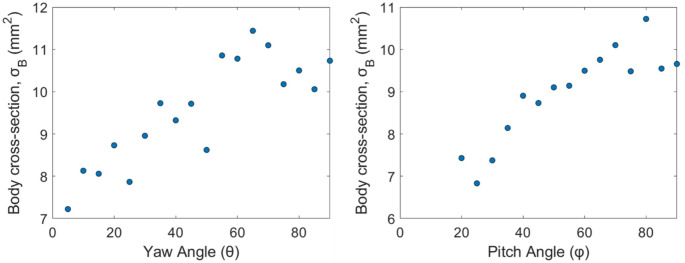
Body optical cross-section ($\:{\sigma\:}_{B}$) as a function of symmetry-reduced yaw ($\:\theta\:$) and pitch ($\:\varphi\:$) angles

The body optical cross-section ($\:{\sigma\:}_{B}$) is determined from the maximum extinction recorded during each segmented transit signal. The $\:{\sigma\:}_{B}\:$values are analyzed with respect to the symmetry-reduced pitch and yaw angles. Figure 10 shows the effect of $\:\theta\:$ and $\:\varphi\:$ angles on body-cross-section measurements. For both orientation components, $\:{\sigma\:}_{B}$ exhibits a clear increasing pattern across the measured range. The body cross section is minimized when the insect is aligned with the laser’s optical axis and maximized when it is perpendicular to it. The measured body cross-section varies from a minimum of 6.8 mm^2^ to a maximum of 11.4 mm^2^.

#### Prediction of symmetry-reduced body orientation

**Fig. 11:**
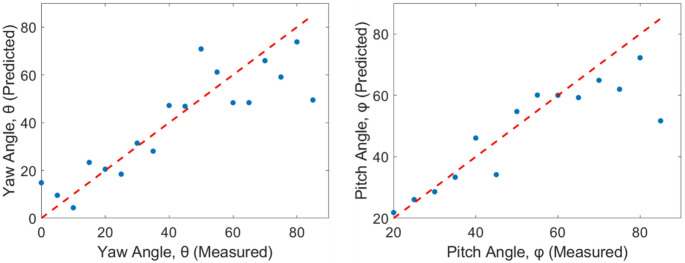
Predicted versus measured insect yaw and pitch angles in the symmetry reduced range

The ability to estimate insect body orientation from optical measurements is evaluated for both symmetry-reduced pitch ($\:\varphi\:$) and yaw ($\:\theta\:$) angles. A Gaussian Process regression model is trained using all MFCCs as input features to predict orientation angles. Model training and evaluation are performed using a 5-fold cross-validation, where 80% data is randomly selected for model training and 20% data is used for testing. This training-testing process repeated 5 times to ensure robustness and generalization of the dataset. The results show good agreement between predicted and measured orientations across the symmetry-reduced range for both pitch and yaw angles. For pitch angle, the model achieves an $\:{R}^{2}=0.75,\:\:p=5.97\times\:{10}^{-6}$ and for the yaw, $\:{R}^{2}=0.76,\:\:p=2.32\times\:{10}^{-6}.$ Fig. 11 shows the predicted orientation angles as a function of measured orientation angles for both yaw and pitch.

#### Correction of body optical cross-section

Since the model is capable of predicting symmetry-reduced orientation ($\:\theta\:$ and $\:\varphi\:$) of an insect, we can apply the orientation effect to correct the insect’s optical body cross-section measurements. A reference orientation at $\:{\theta\:}_{ref}=90^\circ\:$ and $\:{\varphi\:}_{ref}=0^\circ\:$ is chosen to correct all the measured cross-sections, whereas the insect aligns perpendicular to the optical axis. From experimental data, we can compute a correction factor that describes how the insect’s body cross-section changes as a function of both $\:\theta\:$ and $\:\varphi\:$ angles. This correction factor, $\:k$ can be presented by following equation:

$$ k = \frac{{\sigma _{{exp}} \left( {\theta _{{ref}} ,\varphi _{{ref}} } \right)}}{{\sigma _{{exp}} \left( {\theta ,\varphi } \right)}} $$

$$ \sigma _{{corrected}} = k*\sigma _{{measured}} \left( {\theta ,\varphi } \right) $$

**Fig. 12:**
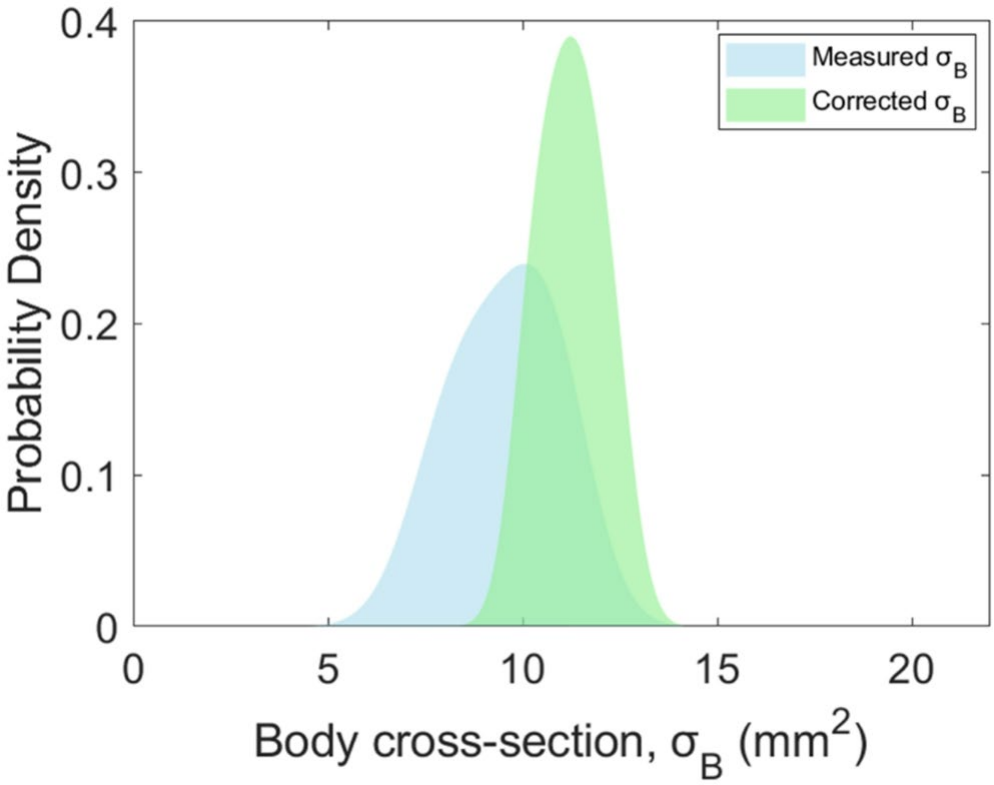
Probability density distribution for measured and corrected optical body cross-section

The measured cross-section multiplied by the corresponding correction factor provides the corrected body cross-section. Figure 12 shows the probability density distribution for both measured and corrected body cross-section. For the measured optical body cross-section, the median value is 9.77 mm^2^ with 1.92 mm^2^ FWHM. The corrected cross-section has a median value of 11.09 mm^2^ and 1.08 mm^2^ FWHM. Hence, the corrected $\:{\sigma\:}_{B}$ provides a more stable measurement of the body size compared to the measured values, increasing the robustness for classification tasks.

## Discussion and conclusion

This study introduced a signal processing framework for quantitatively estimating insect body orientation during flight and correcting the optical cross-section from measured pitch and yaw angles. Our results demonstrate that MFCCs can effectively capture variations in optical transit signals for different orientations.

In the numerical simulation, a 3D flying insect is rotated to understand the orientation effects, which show a significant change by up to 83% in measured body size depending on yaw angles. As seen, the geometric projections of the insect repeat after 180° and mirror after 90° which encourages the symmetry reduction to 0–90°. This angular reduction is a limitation of the single beam photonic system, but it simplifies the prediction task while maintaining physical consistency.

Our laboratory experiment further demonstrates that MFCCs are well suited to characterize the orientation of flying insects during their transit. Combining a field-deployable eBoss sensor with a synchronized camera system, we obtained simultaneous measurements of optical signals from flying insects. MFCCs are extracted from the transit signals, and they show a distinct pattern for both yaw and pitch angles. A non-linear Gaussian Process regression model results in a good agreement between measured and estimated angles with R^2^ = 0.76 for yaw, and R^2^ = 0.75 for the pitch. The predicted angles are used to correct cross-section measurements, which can reduce the uncertainty in morphological and biomass metrics and improve the taxonomic resolution achievable with entomological photonic sensors.

While MFCCs were originally developed for audio signal classification, the combination of simulation-driven modeling and experimental validation further underlines the importance of applying MFCCs in photonic entomology. A key limitation of this approach is that it requires species-specific training data, i.e., laboratory measurements of MFCCs as a function of orientation for each insect of interest, which represents a substantial experimental effort. As a result, direct application to field data remains challenging; nevertheless, these results demonstrate that transit signals do contain orientation information, and that continued advances in data processing and analysis, rather than hardware alone, may lead to significant improvements in taxonomic accuracy. MFCCs provide a reliable feature set for estimating body angles, correcting orientation-dependent features, and refining biomass calculations. Together, these improvements enhance the performance of photonic sensors and strengthen their potential for species classification and ecological monitoring.

## Data Availability

No datasets were generated or analysed during the current study.
